# Identification and Characterization of Marine Microorganisms by Tandem Mass Spectrometry Proteotyping

**DOI:** 10.3390/microorganisms10040719

**Published:** 2022-03-26

**Authors:** Clément Lozano, Mélodie Kielbasa, Jean-Charles Gaillard, Guylaine Miotello, Olivier Pible, Jean Armengaud

**Affiliations:** Département Médicaments et Technologies pour la Santé (DMTS), Université Paris-Saclay, CEA, INRAE, SPI, F-30200 Bagnols-sur-Cèze, France; clement.lozano@cea.fr (C.L.); melodie.kielbasa@cea.fr (M.K.); jean-charles.gaillard@cea.fr (J.-C.G.); guylaine.miotello@cea.fr (G.M.); olivier.pible@cea.fr (O.P.)

**Keywords:** phylopeptidomics, proteomics, microorganisms, tandem mass spectrometry, proteotyping, identification, molecular phenotyping

## Abstract

The vast majority of marine microorganisms and their functions are yet to be explored. The considerable diversity they encompass is an endless source of knowledge and wealth that can be valued on an industrial scale, emphasizing the need to develop rapid and efficient identification and characterization techniques. In this study, we identified 26 microbial isolates from coastal water of the NW Mediterranean Sea, using phylopeptidomics, a cutting-edge tandem mass spectrometry proteotyping technique. Taxonomical identification at the species level was successfully conducted for all isolates. The presence of strains belonging to the newly described Balneolaeota phylum, yet uncharacterized at the proteomics scale, was noted. The very first proteomics-based investigation of a representative of the Balneolaeota phylum, *Balneola vulgaris*, is proposed, demonstrating the use of our proteotyping workflow for the rapid identification and in-depth molecular characterization, in a single MS/MS analytical run. Tandem mass spectrometry proteotyping is a valuable asset for culturomic programs as the methodology is able to quickly classify the most atypical isolates.

## 1. Introduction

Marine bacteria are the key to major environmental processes. Their role in biogeochemical cycles [1], symbiotic interactions [2], and bioremediation [3] has been documented. Their potential as a source of new therapeutics and biotechnological applications is considered to be highly promising and has given rise to blue biotechnology. In the last two decades, the emergence of proteomics allowed the understanding of the bacterial response toward environmental stressors [4], pollutants [5], and antibiotics [6], providing an insight into the bacterial functional response. Proteomics and metaproteomics are considered the best molecular approaches to explore the functioning of microorganisms, from pure culture to complex microbial communities [7]. Their recent advances in terms of methodology [8] and possible applications in the field of blue biotechnology [9] have been reviewed.

Proteotyping is a powerful tool to identify microorganisms from various samples. The emergence of mass spectrometry in the field of diagnostics, especially via MALDI-TOF, has revolutionized bacteriology, previously dominated by molecular techniques based on DNA amplification. Because MALDI-TOF-based identification requires a comprehensive spectral database in order to identify the microorganisms present in the tested samples, this technology is not suitable for environmental samples and mixtures of microorganisms. As tandem mass spectrometry delivers more information than MALDI-TOF, alternative proteotyping approaches have been developed based on the identification of discriminating peptides or peptidome similarities [10,11,12]. Such technology requires no a priori, and is amenable to the high-throughput level required for characterizing numerous isolates from culturomics [13].

Phylopeptidomics, a novel proteotyping concept first described in Pible et al. [14], allows for the taxonomic identification and relative biomass quantification of microorganisms in a sample [15], based on the combination of common and taxa-specific peptides obtained via shotgun proteomics. This technique presents numerous advantages as it relies on public databases of annotated sequenced genomes and requires no experimental spectral database. If a closely related genome is present in the database, it allows—in a single run—the identification of any prokaryotic and eukaryotic species, unlike 16S targeted sequencing approaches.

In the present study, we conducted—via state-of-the-art mass UPLC-MS/MS—the phylopeptidomics-based proteotyping of 26 marine bacteria, using a validated experimental workflow already documented as being robust and reliable for bacterial identification [13]. Further proteomic functional analyses were conducted for two environmentally relevant bacteria to exemplify the wealth of information given by a single analytical run. To our knowledge, this is the first application of tandem mass spectrometry proteotyping for the identification of marine microbial isolates, and the characterization of their respective proteomes, using a workflow fitting a 48 h window.

## 2. Materials and Methods

### 2.1. Sampling and Bacterial Isolation

Seawater was collected in a clean plastic bottle in Saintes-Maries-De-La-Mer (Northwestern Mediterranean sea, France, 43°26′54.3” N 4°25′03.4” E). Samples were kept at room temperature in the dark for 12 months before isolation on agar plates using the dilution to extinction method. Bacterial isolates were cultured in marine broth 2216 (DIFCO) at 25 °C. Aliquots were kept at −80 °C in glycerol saline buffer containing 21% glycerol, 0.05 M MgSO_4_, and 0.025 M Trizma base until further use.

### 2.2. Bacterial Culture and Protein Extraction

Bacteria were cultured in 7 mL of marine broth at 25 °C in 15 mL tubes until turbidity appeared (24 h to 48 h). Liquid cultures were centrifuged for 15 min at 8000× *g*. Supernatants were discarded and cell pellets were kept at −20 °C until use. Proteins from the cell pellets were extracted as described in Hayoun et al. [16]. Briefly, pellets were suspended in 200 µL of LDS buffer containing 26.5 mM Tris HCl, 35.25 mM Tris base, 0.5% LDS, 2.5% Glycerol, and 0.13 mM EDTA, supplemented with 5% beta-mercaptoethanol. Samples were incubated for 5 min at 99 °C in a thermomixer (Eppendorf, Hamburg, Germany) and sonicated for 5 min in an ultrasonic water bath (VWR ultrasonic cleaner). Samples were transferred into 2 mL Screw Cap microtubes (Sarstedt, Nümbrecht, Germany) containing 200 mg of beads. Bead beating was performed with a Precellys Evolution instrument (Bertin Technologies, Rockville, MD, USA) at 10,000 rpm for 10 cycles of 30 s, with 30 s of pause between each cycle. Samples were centrifuged at 16,000× *g* for 1 min and supernatants were transferred to new microcentrifuge tubes before incubation at 99 °C for 5 min.

### 2.3. Single-Pot Solid-Phase-Enhanced Sample Preparation (SP3) Proteolysis

SP3 digestion, first described by Hughes et al. [17], was performed in a 96-well plate as described in Hayoun et al. 2019 [13]. A 1/1 mix of hydrophilic (Ref. *n*°24152105050250) and hydrophobic (Ref. *n*°44152105050250) Sera-Mag™ Magnetic beads (Merck) at 50 mg/mL was prepared and stored at 4 °C until use. A total of 200 μg of beads (4 μL) was added to 20 μL of cell lysate. The mix was acidified by adding 12 μL of formic acid and beads were activated with 204 μL CH_3_ CN (85% final concentration). Bead–protein complexes were trapped using MagnaBind (Thermo Scientific, Waltham, MA, USA). Supernatants were discarded and proteins were washed twice with 200 μL of 70% ethanol and once with 180 μL CH_3_CN. Proteins were digested at 37 °C for 30 min with 50 μL of digestion buffer containing 0.1 μg of Trypsin Gold (Promega, Madison, WI, USA) in 50 mM NH_4_HCO_3_, supplemented with 0.01% of ProteaseMAX surfactant (Promega). Beads were trapped as described above and the resulting peptides were acidified with trifluoroacetic acid (final concentration 0.5%) before LC MS/MS analysis.

### 2.4. UPLC-MS/MS

Peptides were analyzed with a Q-Exactive HF (Thermo Scientific) tandem mass spectrometer coupled to an ultimate 3000 nano-LC system (Thermo Scientific). Peptides were desalted on a reverse-phase PepMap 100 C18 μ-precolumn (5 mm, 100 Å, 300 mm i.d. × 5 mm, Thermo Scientific) and separated on a nanoscale PepMap 100 C18 nanoLC column (3 mm, 100 Å, 75 mm i.d. × 50 cm, Thermo Scientific) at a flow rate of 0.3 μL/min using a 30 min gradient (2.5% B from 0 to 1.5 min, 2.5–25% B from 1.5 to 26.5 min, and 25–40% B from 26.5 to 30 min) of mobile phase A (0.1% HCOOH/100% H_2_O) and phase B (0.1% HCOOH/80% CH_3_CN). The mass spectrometer operated in data-dependent acquisition mode with a Top20 strategy, i.e., the 20 most abundant precursor ions were serially selected for fragmentation. Full-scan mass spectra were acquired from 350 to 1800 m/z. Only peptides with 2 or 3 positive charges were selected for fragmentation with a dynamic exclusion time of 10 s and an isolation window of 1.6 m/z.

### 2.5. Proteotyping

Proteotyping-based identification was conducted using an in-house-developed procedure consisting of a cascade search as follows: (1) the 10,000 best spectra were selected to run a Mascot search against a subset of the NCBInr database containing one representative per species and including 94,176,939 protein sequence entries totaling 39,636,215,241 amino acids and corresponding to 50,995 organisms (494 Archaea, 2231 Eukaryota, 12,047 Bacteria, and 36,223 Viruses); (2) all spectra were used for a Mascot query against a database reduced to the genera previously identified during step 1, and all their descendants; (3) similarly, all spectra were searched for against a database reduced to the species identified during step 2. Peptides were validated using a *p*-value below 0.3, 0.15, and 0.05 for steps 1, 2, and 3, respectively. Mascot searches were set up as follows: 3 ppm peptide tolerance during step 1, and 5 ppm peptide tolerance during steps 2 and 3, 0.02 Da MS/MS fragment tolerance, 2+ or 3+ peptide charges, a maximum of two missed cleavages, carbamidomethylation of cysteine as fixed modification, oxidation of methionine as variable modification, and trypsin as a proteolytic enzyme.

### 2.6. Proteomics and Functional Characterization

Protein identification was performed using Mascot Daemon software version 2.6.1 (Matrix Science) with the same parameters as described above. Each dataset was queried using a dedicated genus-specific database [18] (taxid 662 for *Vibrio* and 455358 for *Balneola*). Proteins identified at a false-discovery rate below 1% were further considered and annotated using KEGG Orthology (KO) terms via GhostKOALA (Available online: https://www.kegg.jp/ghostkoala/ accessed on 18 February 2022) [19].

### 2.7. Mass Spectrometry Data

The mass spectrometry and proteomics dataset are available through the ProteomeXchange Consortium via the PRIDE partner repository (Available online: https://www.ebi.ac.uk/pride/ accessed on 18 February 2022), under dataset identifiers PXD031583 and 10.6019/PXD031583.

## 3. Results

### 3.1. Identification of Microbial Isolates

Seawater was sampled and kept at room temperature in the dark without agitation for 12 months in order to select under-represented marine taxa that could survive this condition and could be more challenging to identify than more abundant marine microorganisms. Isolation on marine medium agar plate resulted in 26 isolates. The bacterial identifications, obtained by tandem mass spectrometry, are shown in Table 1. MS/MS spectra number, along with the percentage of these spectra assigned to a peptide sequence, i.e., the peptide-to-spectra-matches (PSMs), and the number of PSMs assigned to a taxon at the species level, are provided. On average, 19,527 ± 1310 MS/MS spectra were recorded within 30 min, and 56.9 ± 3.8% were attributed to peptide sequences. This constant and high assignment rate indicates a good quality of the MS/MS spectra recorded for all samples. The number of PSMs attributed to a taxon, known as taxon-to-spectra-matches (TSMs) as previously defined [14], ranged from 6610 to 11,871. This number may be influenced by the density of genome sequences available for each taxon and the coverage of the genome sequence diversity within this taxon and its closely related neighbors. For environmental samples, especially marine isolates, this coverage is rather low, with a few representatives per species. Here, we introduced a novel ratio that corresponds to the percentage of PSMs assigned to TSMs of the identified species. This ratio is between 78.5 and 99.5% at the species level for the present dataset with an average of 96.4 ± 5.3%. The ratio depends on the relationship between the isolate and the reference genomes present in the database. A lower value may indicate that the sample harbors a significant number of peptide sequences that are not present in the reference genome but present in other representatives, which may not have been sequenced and therefore absent from the database. The lowest TSMs/PSMs ratio values were obtained for the isolates 4A (78.5%) and 8 (82.9%), both assigned to *Thalassospira profundimaris*. Notably, the last round database comprises eight genomes uncovering eight different species in the *Thalassospira* genus: *Thalassospira profundimaris, Thalassospira lucentensis, Thalassospira lohafexi, Thalassospira australica, Thalassospira xiamenensis, Thalassospira alkalitolerans, Thalassospira marina,* and *Thalassospira mesophila*. At the genus level, the TSMs/PSMs ratio was 91.0% and 95.9% for the two samples, respectively. Altogether, these results suggest that these isolates most likely belong to unsequenced *Thalassospira* species that share a significant number of protein sequences between several species.

**Figure 1:**
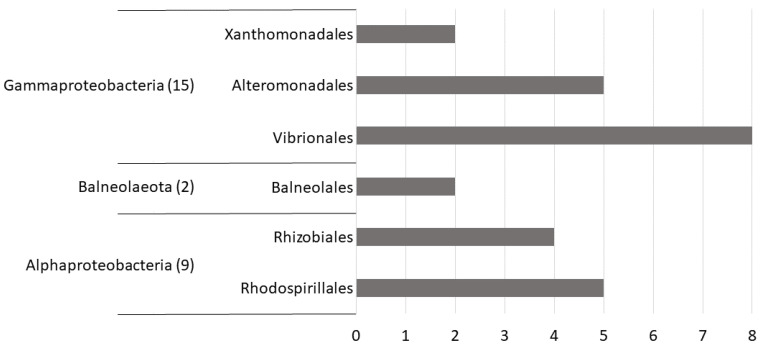
Diversity of bacterial isolates at the phylum and order level.

As illustrated in Figure 1, among the 26 identified bacteria, 15 belong to Gammaproteobacteria, nine are Alphaproteobacteria, and two are Balneolaeota. At the order level, Vibrionales dominate with eight isolates, followed by Rhodospirallales, Alteromonadales, Rhizobiales, Xanthomonadales, Balneolales, and Sphingomonadales with 5, 5, 4, 2, and 2 isolates, respectively (Figure 1). Although Firmicutes, Actinobacteria, and Planctomycetes are absent from this list due to the low sample number, these observations are environmentally relevant considering the Proteobacteria dominance in marine waters [20].

### 3.2. Examples of Phylopeptidomic Signatures

**Figure 2:**
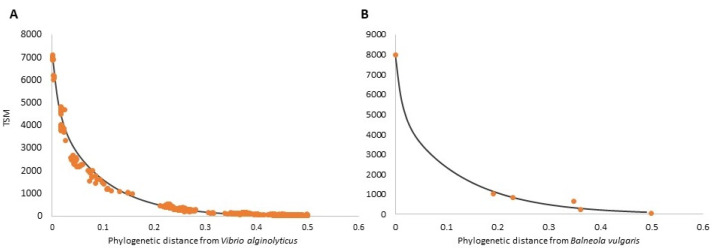
Phylopeptidomic signatures and TSM values assigned to each taxon in the database as a function of the phylogenetic distance from *Vibrio alginolyticus* (**A**) and *Balneola vulgaris* (**B**). Orange dots represent experimentally obtained TSMs by querying the full NCBInr database while the black curve represents the modelized phylopeptidomic signature, i.e., a theoretical exponential distribution of TSMs relative to the phylogenetic distance separating organisms present in the queried database.

Phylopeptidomic signatures of *V. alginolyticus* (sample 25B) and *Balneola vulgaris* (sample 5) are shown in Figure 2A,B, respectively. These signatures represent the mathematic fit explaining the number of TSMs as a function of the phylogenetic distance to the identified taxon. Although TSMs contributing to the signature (d < 0.5) can be assigned to 5180 sequenced organisms phylogenetically distant from *V. alginolyticus* (Figure 2A), only six organisms were found in the database for *B. vulgaris* (Figure 2B). This discrepancy, due to a high number of fully sequenced Vibrionales compared to the newly described Balneolales [21], reminds us that proteotyping relies on public databases. Hence, identification accuracy depends on the taxonomic depth of the queried database and its annotation quality [22,23].

Despite the low density of sequenced Balneolaeota in the database (two genomes), we noted a remarkably high level of TSMs assigned to *B. vulgaris* [24], indicating close phylogenetic proximity between the isolate 5 and the sequenced *B. vulgaris* strain present in the queried database. In future, increasing efforts at sequencing additional Balneolaeota representatives may improve the fit presented in Figure 2B.

### 3.3. Proteome Characterization of Two Environmentally Relevant Marine Bacteria

**Figure 3:**
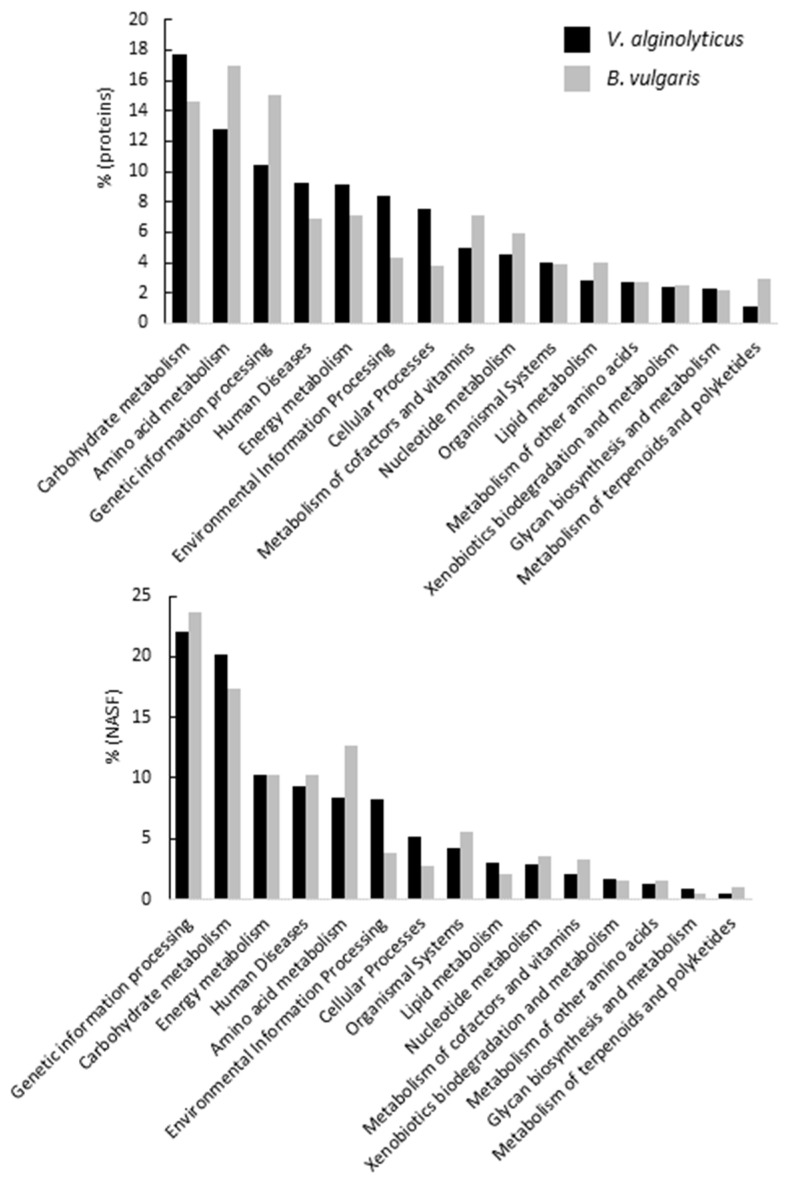
KEGG functional characterization of the proteomes of *Vibrio alginolyticus* and *Balneola vulgaris* using protein functional attribution (**top** panel) and functional attribution weighted by the NSAF of each protein (**bottom** panel).

Two bacterial species were used as an example to illustrate the ability of our workflow to provide a comprehensive proteomic analysis for each bacterium. The acquired MS/MS spectra dataset was exploited by means of a shotgun pan-proteomics workflow [18]. The complete proteomes of *V. alginolyticus* (sample 25B, Table S1) and *B. vulgaris* (sample 5, Table S2) comprise a total of 1173 and 1138 identified proteins, uncovering 25.8% and 46.8% of the theoretical proteomes, respectively. Notably, proteome coverages could can be optimized by multiplying culture conditions and bacterial physiological states [4,25]. The list of proteins was validated at a false discovery rate set to 1% and the abundance of each protein was assessed by their respective spectral counts. Protein annotation was conducted using KO terms, although ontology-based annotation is described to be perfectible, especially for prokaryotic species [26]. Figure 3 reports the percentage of proteins identified for each main functional category for both isolates (left panel) and the protein biomass for each of these categories (right panel). For the latter estimation, protein abundances were weighted using normalized spectral abundance factor (NSAF) as defined [27]. Although “carbohydrate metabolism” was the dominating function, representing 17.7% for *V. alginolyticus*, it held third place and accounted for 14.6% for *B. vulgaris*, for which the greatest function was “amino acid metabolism”, with 17.0%. Function weighting using NSAF provides a more representative picture of the protein abundance by function. “Genetic information processing” becomes the most abundant KO term, followed by “carbohydrate metabolism”, for both species. The percentages of molecules devoted to “amino acid metabolism” in both species differ drastically: 12.7% of the identified proteins are involved in this process in *B. vulgaris* whereas only 8.4% are involved in *V. alginolyticus.* Major differences are also observed for proteins involved in “environmental information processing”, with 8.2% of the protein biomass for V. *alginolyticus* but only 3.9% for *B. vulgaris*. These discrepancies may be related to fundamental differences in their adaptation to their natural environment.

The comparison of the number of identified proteins belonging to particular functions between *V. alginolyticus* (isolate 25B) and *B. vulgaris* (isolate 5) revealed major differences between the two species (Table 2). Particularly interesting for preliminary functional molecular characterization, the functions Beta-lactam resistance and Biofilm formation were selected as they may be valuable in clinical microbiology, whereas Flagellar assembly, Bacterial chemotaxis, and Quorum sensing are at the core of recent environmental microbiology studies [28,29]. The proteome of the former harbored 40 proteins involved in Biofilm formation, 23 in Quorum sensing, 16 in Chemotaxis, seven in Flagellar assembly and 10 in Beta-lactam resistance. On the other hand, *B. vulgaris* held only six proteins involved in biofilm formation, 15 in quorum sensing and none in chemotaxis, suggesting that this bacterium is non-motile and non-biofilm forming in our culture condition.

## 4. Discussion

Cost-effective, fast, and reliable identification of microorganisms constitutes a challenging task, especially for environmental strains, the majority of which are unsequenced and poorly studied. As previously documented, the phylopeptidomics approach used in this study permits the identification at the species level with no a priori data required for any isolate regardless of its eukaryotic or prokaryotic nature [14,30].

We successfully identified 26 environmental marine isolates (Table 1), most of them belonging to the Gammaproteobacteria, followed by Alphaproteobacteria. We also identified two isolates belonging to Balneolaeota (Figure 1), a new phylum recently separated from the Bacteroidetes [21], which have been poorly investigated to date. Here, we identified *Balneola vulgaris*, a species first isolated from the bay of Banyuls-sur-Mer in the north-western Mediterranean Sea and described in 2006 [24]. The present new isolate was from sampling undertaken by the seaside 200 km away and obtained with the same culture conditions. Information on this species is scarce and limited to morphological and phenotypic characterizations [31,32]. We provided its first description at the molecular level, its genome sequence being available since 2013 through a program performed by the DOE Joint Genome Institute but until now not explored. Proteomic characterization suggests that *V. alginolyticus* is capable of quorum sensing, biofilm formation, and motility, which is supported by the literature [33,34,35]. Our data suggest that *B. vulgaris* is non-motile and does not harbor a flagellum, as described in a prior study [24]. When taking into account the NSAF, we observed that the percentage attributed to each function changed significantly (Figure 3, bottom panel), providing a better picture of the proteome of our isolates in the present culture conditions. Interestingly, we observed for both strains an increase in the attribution percentage of proteins involved in Genetic information, Carbohydrate metabolism, and Energy metabolism, which is consistent considering that both bacteria were cultured in a rich medium. A total of 10 and 7 proteins were attributed to Beta-lactam resistance for *V. alginolyticus* and *B. vulgaris*, respectively. Among these proteins, *B. vulgaris* harbored an efflux RND transporter periplasmic adaptor subunit (WP_157464801.1), a penicillin-binding protein 1A (WP_018126279.1), and the outer membrane protein TolC (WP_018126232.1), documented to be involved in antibiotic export and resistance to antimicrobial peptides [36,37,38].

We demonstrated that an MS/MS proteotyping method such as phylopeptidomics allows, in a single run, the identification at the species level and the proteomic characterization of environmental isolates, corroborating a considerable advantage over conventional techniques such as MALDI-TOF or PCR. Notably, phylopeptidomics applies well to culturable isolates as these microorganisms have currently more chances to be genome sequenced. However, metagenome-assembled genomes can also be used to define new species and will quickly populate genome and taxonomy databases [39], and phylopeptidomics can theoretically be applied to more direct analysis of seawater without the need for cultivation. In addition, we highlighted the capability of the technique for the detection of proteins involved in antibiotic resistance, which is an asset for clinical and environmental sciences. Thus, phylopeptidomics may be a valuable asset for culturomic programs because the methodology is able to quickly classify the most atypical isolates. We provided the first proteomic characterization of a bacterial species belonging to the Balnoleata phylum and emphasized a gap in the literature that may inspire future sequencing efforts.

## Figures and Tables

**Table 1:** Proteotyping results for the 26 bacterial isolates ranked by alphabetical order, including the species taxonomy and MS/MS spectra assigned characteristics.

Sample ID	MS/MS Spectra	% of MS/MS Spectra Resulting in PSMs	Species	Species-Assigned TSMs	TSMs/PSMs Ratio (%)
4D	13508	50.6	*Balneola vulgaris*	6610	96.7
5	19600	59.7	*Balneola vulgaris*	11578	98.9
12	18645	58.1	*Marinobacter salarius*	10713	98.8
6B	20211	62.6	*Marinobacter salarius*	12553	99.2
7	19994	57.9	*Marinobacter salarius*	11441	98.8
1B	20109	50.5	*Martelella mediterranea*	9208	90.6
19	19612	59.4	*Ochrobactrum pseudogrignonense*	10402	89.3
13	20115	59.2	*Ochrobactrum pseudogrignonense*	11786	98.9
15B	19502	61.7	*Ochrobactrum pseudogrignonense*	11871	98.7
17	18259	58	*Pseudoalteromonas piscicida*	10377	97.9
21	19533	55.6	*Pseudoalteromonas piscicida*	10694	98.5
14	19475	58.5	*Stenotrophomonas maltophilia*	11331	99.5
18B	19882	57.4	*Stenotrophomonas maltophilia*	11347	99.4
10	20355	56.4	*Thalassospira lucentensis*	10961	95.5
8	20249	47	*Thalassospira profundimaris*	7894	82.9
4A	20053	48.6	*Thalassospira profundimaris*	7650	78.5
3	19955	59.6	*Thalassospira xiamenensis*	11755	98.8
1A	19681	60.8	*Thalassospira xiamenensis*	11542	96.4
16	19769	57.2	*Vibrio alginolyticus*	11136	98.5
20	20090	57.9	*Vibrio alginolyticus*	11454	98.5
22	19780	57.4	*Vibrio alginolyticus*	11201	98.6
23	19748	56.7	*Vibrio alginolyticus*	11039	98.6
24	20011	57.4	*Vibrio alginolyticus*	11321	98.6
18A	19990	56.8	*Vibrio alginolyticus*	11160	98.3
23A	19632	56.8	*Vibrio alginolyticus*	11006	98.7
25B	19945	56.8	*Vibrio alginolyticus*	11112	98.2

**Table 2:** Comparison of the numbers of identified proteins belonging to major functions between *V. alginolyticus* and *B. vulgaris*.

Functions	Number of Identified Proteins	
	*V. alginolyticus* (Isolate 25B)	*B. vulgaris* (Isolate 5)
Beta-lactam resistance	10	7
Quorum sensing	23	15
Bacterial chemotaxis	16	0
Biofilm formation	40	6
Flagellar assembly	7	1

## Data Availability

Not applicable.

## Supplementary Materials

The following supporting information can be downloaded at: https://www.mdpi.com/article/10.3390/microorganisms10040719/s1, Table S1: Identified proteins of *V. alginolyticus* (sample 25b) and their spectral counts; Table S2. Identified proteins of *B. vulgaris* (sample 5) and their spectral counts.
